# Factors Affecting the Aluminum, Arsenic, Cadmium and Lead Concentrations in the Knee Joint Structures

**DOI:** 10.3389/fpubh.2021.758074

**Published:** 2021-12-23

**Authors:** Guoyong Li, Chunfeng Xiong, Wenhua Xu, Runhong Mei, Tao Cheng, Xuefeng Yu

**Affiliations:** ^1^Department of Orthopaedics, The Fourth Affiliated Hospital of Nanchang University, Nanchang, China; ^2^Department of Urology, Jiangxi Children's Hospital Affiliated to Nanchang University, Nanchang, China; ^3^Department of Orthopaedics, Yichun People's Hospital, Yichun, China; ^4^Department of Orthopaedics, Shanghai Jiao Tong University Affiliated Sixth People's Hospital, Shanghai, China

**Keywords:** aluminum, arsenic, cadmium, lead, knee joint, structures, factors

## Abstract

**Background:** Toxic elements, such as aluminum (Al), arsenic (As), cadmium (Cd), and lead (Pb), are persistent environmental pollutants that can cause adverse effects on the health of exposed individuals. Bone is one of the primary target organs of accumulation and potential damage from toxic elements.

**Objectives:** This study was performed to determine the Al, As, Cd, and Pb concentrations in the femoral cancellous bone, femoral cartilage, anterior cruciate ligament, meniscus, tibial cartilage, tibial cancellous bone and infrapatellar fat pad. Furthermore, the aim of this study was to explore the relationships between toxic element concentrations and related factors such as gender, age, place of residence, hypertension and diabetes, and to determine the correlations among these toxic elements in knee joint structures.

**Methods:** The samples used this study were collected from 51 patients following total knee arthroplasty. The Al, As, Cd, and Pb concentrations were determined using inductively coupled plasma optic emission spectrometry.

**Results:** Significant differences were found in the Al, As, Cd, and Pb concentrations among the knee joint structures. Cd concentration in the tibial cancellous bone in women was significantly higher than in men. Pb concentration in the infrapatellar fat pad of urban patients was significantly higher as compared to rural patients. Al concentrations in the femoral cancellous bone, femoral cartilage, anterior cruciate ligament, meniscus and tibial cartilage were significantly higher in patients living in urban areas than in rural areas. As concentration in the tibial cancellous bone of diabetic patients was significantly higher compared to non-diabetic patients. In addition, significant Spearman's positive correlations were found between Al and Pb in the knee joint structures.

**Conclusion:** The obtained results of the investigated toxic elements may serve as a basis for establishing the reference values of Al, As, Cd, and Pb in the knee joint structures. The results reported in the study provides novel data regarding the relationships between the toxic element concentrations and gender, age, place of residence, hypertension and diabetes in the studied structures of knee joint. Furthermore, new interactions among these toxic elements were noted.

## Introduction

Toxic elements are worldwide environmental pollutants with various ill-health effects (1). Environmental and occupational exposure to toxic elements has become a major public health concern. Human beings are exposed to toxic elements from multiple sources, such as contaminated food, air, drinking water and soil, as well as occupational exposure, which are usually absorbed into the body through inhalation, dermal and ingestion routes (2, 3). These toxic elements interfere with homeostasis in the organism and pose serious threats to human health (4). We are challenged by an increasing necessity of monitoring not only environmental pollution but also the toxic element concentrations in human tissues (5). Due to the characteristics and long-term remodeling of bone, it may reflect chronic exposure to the toxic elements and serve as a basis for the indirect assessment on the degree of environmental pollution (5–8).

Al is integrated into the bone matrix with a half-life of 10–20 years (9, 10). Al deposition in bone inhibits bone mineralization by reducing the calcium, magnesium, and phosphorus levels (11). In addition, Al can decrease collagen synthesis, reduce bone formation and damage bone remodeling (12), which can lead to bone diseases, such as renal osteodystrophy (13) and osteomalacia (14). As has been related to the decreased osteoblast proliferation and increased osteoclasts multiplication (15), possibly due to the As-induced reduction in transcription factor expression such as bone morphogenetic protein-2 and osteocalcin. Such results lead to alterations of cortical and trabecular bone microarchitecture (16, 17) and reduce bone mineral density and trabecular bone volume (18). Clinical epidemiological trials have demonstrated the correlation between As poisoning and Paget's disease (19). Bone is one of the primary target organs of Cd toxicity in the body (20). The reduction of bone mineral density is closely associated with Cd pollution in the environment (21). Cd can directly affect bone-related cell activity, which accelerates bone resorption, inhibits bone formation, destroys bone microstructure, and eventually causes bone injury (20, 22). Long-term exposure to Cd can lead to bone metabolic diseases, such as itai-itai disease (23) and osteoporosis (20). Pb exhibits the high affinity for bone due to its ability to replace other divalent cations, such as calcium and magnesium ions in the body (24). Studies have demonstrated that Pb exposure is associated with decreased bone density, bone growth retardation, osteoporosis and osteoarthritis (25, 26). Oxidative stress is considered as one of the main factors in Pb-induced bone damages because of the production of reactive oxygen species induced by Pb exposure (27). In addition, Pb inhibits osteoblast activity and induces osteoblast apoptosis by controlling the Wnt signaling pathway (28).

To our knowledge, there is limited data on the As concentration in the knee joint and the available information concerning the Al, Cd, and Pb concentrations is insufficient (Table 1). Studies focusing on the Al, As, Cd, and Pb concentrations in joints were performed mainly in the Polish population (Table 1). The distributions of toxic elements in the environment differ across regions (42). Hence, this study was performed to determine the Al, As, Cd, and Pb concentrations in the knee joint in Chinese population. The aim of this study was to explore the relationships between the toxic element concentrations and biological factors, environmental factors and health status, and to examine the correlations among these toxic elements in the studied structures of knee joint.

**Table 1:** Mean concentrations of Al, As, Cd, and Pb in the cartilage, cancellous bone, anterior cruciate ligament, meniscus and infrapatellar fat pad in patients from various countries, based on literature data.

**Author**	**Country**	**Publication year**	**Samples (*n*)**	**Al**	**As**	**Cd**	**Pb**
**Cartilage**							
Brodziak-Dopierala et al. (29)	Poland	2015	91			0.81	
Brodziak-Dopierala et al. (30)	Poland	2011	53				3.03
Brodziak-Dopierala et al. (31)	Poland	2006	110			0.26	3.97
Jurkiewicz et al. (32)	Poland	2005	45				3.53
Kosik-Bogacka et al. (33)	Poland	2018	30				1.9
Lanocha et al. (34)	Poland	2013	37			0.031	0.527
Lanocha et al. (5)	Poland	2012	30			0.022	0.41
**Cancellous bone**							
Babuśka-Roczniak et al. (35)	Poland	2020	50	55.2			
Brodziak-Dopierala et al. (29)	Poland	2015	91			0.94	
Brodziak-Dopierala et al. (30)	Poland	2011	53				1.90
Brodziak-Dopierala et al. (31)	Poland	2006	110			0.46	2.70
Jurkiewicz et al. (32)	Poland	2005	45				2.56
Jurkiewicz et al. (36)	Poland	2004	38		0.32	0.047	2.05
Kuo et al. (37)	China	2000	70	52.1	3.6	1.2	7.1
Lanocha-Arendarczyk et al. (38)	Poland	2015	33			0.05	1.87
Lanocha et al. (34)	Poland	2013	37			0.028	0.500
Lanocha et al. (5)	Poland	2012	30			0.035	0.49
Roczniak et al. (39)	Poland	2017	50			0.016	
Roczniak et al. (40)	Poland	2017	50				2.67
Stojsavljević et al. (3)	Serbia	2019	25		8.8	0.014	0.91
Zaichick et al. (7)	Russia	2011	80	7.2		0.044	2.24
Zioła-Frankowska et al. (4)	Poland	2015	96				1.15
**Anterior cruciate ligament**							
Kosik-Bogacka et al. (33)	Poland	2018	30				0.1
**Meniscus**							
Babuśka-Roczniak et al. (35)	Poland	2020	50	31.3			
Kosik-Bogacka et al. (33)	Poland	2018	30				0.1
Roczniak et al. (39)	Poland	2017	50			0.010	
Roczniak et al. (40)	Poland	2017	50				0.32
**Infrapatellar fat pad**							
Żaneta et al. (41)	Poland	2019	46				0.51

*Al, As, Cd, and Pb concentrations are expressed as mg/kg dry weight*.

## Materials and Methods

### Subject Selection

This study was conducted with the approval of Ethics Committee of Shanghai Sixth People's Hospital No. 2021-KY-022(k). A total of 51 participants aged 54–81 years were involved in this study. Thirty-five cases were women aged 54–81 years and the remaining 16 cases were men aged 56–79 years. The indication of total knee arthroplasty was the knee degenerative disease with severe dysfunction and chronic pain. The patients signed the written consent form to participate in this study and were surveyed regarding their demographics and health status, with special emphasis on factors that possibly affected the toxic element concentrations in the knee joint. Participants were grouped according to age, gender, place of residence, hypertension and diabetes. All samples, including 51 samples of femoral cancellous bone, femoral cartilage, anterior cruciate ligament, meniscus, tibial cartilage, tibial cancellous bone and infrapatellar fat pad, were obtained from 51 patients following total knee arthroplasty. The samples were marked with codes and stored in modified polyethylene containers in a refrigerator at a temperature of −22°C.

### Mineralization and ICP-OES Analysis

The knee samples were prepared according to the study of Kuo et al. (37) with some modifications. The research materials were immersed into acetone for 1 h to remove the lipid and were cleaned by ultrapure water (Integral V, milliQ, USA). Subsequently, the sample was dried in an incubator at 105°C until no further weight reduction occurred. Tissue samples with a known mass (0.2 g) were mineralized using 5 ml of concentrated 65% nitric acid (Suprapure Merck, Darmstadt, Germany) and 1 ml of 30% hydrogen peroxide (Baker Analyzed, Phillipsburg, NJ, USA) in a microwave digestion system (ETHOS One, Milestone, Italy). Mineralization was a two-stage procedure. The first stage lasted 10 min at 140°C, whereas the second stage was 30 min at 180°C. The post-mineralization solution was diluted to the milliliter mark with ultrapure water.

The determination of Al, As, Cd, and Pb concentrations in mineralized samples were performed adopting inductively coupled plasma optic emission spectrometry (ICP-OES, Agilent 5100, USA) (43). The operating parameters were as follows: RF power, 1.0 kW; plasma flow, 12 L/min; auxiliary flow, 1.0 L/min; pump rate, 12 rpm; emission lines of Al: λ = 396.152 nm, As: λ = 188.980 nm, Cd: λ = 226.502 nm, Pb: λ = 220.353 nm. The calibration curve method was adopted. The standard solutions of 0.1 mg/ml (Inorganic Ventures, USA) as well as ultrapure water were applied. The results correspond to the average concentrations obtained for all analytical lines of the toxic elements, with standard deviation of not higher than 1.5%. The National Institute of Standards and Technology-Standard Reference Material (NIST-SRM) 1486 bone meal was used to validate the accuracy of the analytical procedure. The concentration values of standard reference materials were provided by the manufacturers and our measured values were presented in Table 2.

**Table 2:** Analysis of NIST-SRM 1486 (National Institute of Standards and Technology-Standard Reference Material) bone meal (mg/kg dry weight).

**Chemical elements**	**Certified**	**Measured (*n* = 8)**	**Recovery (%)**
Al	<1	1.173 ± 0.091	–
As	0.006	0.0063 ± 0.0012	105
Cd	0.003	0.0025 ± 0.0003	83.3
Pb	1.335	1.291 ± 0.106	96.7

### Statistical Analysis

The statistical analysis was performed with IBM SPSS Statistics, version 26.0 (IBM Corp., Armonk, NY). The normal distribution of data was not confirmed after applying the Shapiro-Wilk test. Statistical analysis utilized non-parametric tests. Intergroup comparisons were performed using the Mann-Whitney U test. The Kruskal-Wallis test was used for the comparisons of multiple groups. Moreover, the Spearman's rank correlations between these toxic elements occurring in the knee joint structures were determined. The differences were considered statistically significant at *p* < 0.05.

## Results

The concentrations of the toxic elements in almost all of the analyzed structures of knee joint were in descending order as follows: Al>As>Pb>Cd (Table 3). Al concentration was the highest in the infrapatellar fat pad. As concentration in the meniscus was by far higher than in the femoral cancellous bone (Table 3). Significant differences were observed in the concentrations of Al, As, Cd, and Pb among the knee joint structures (Table 3).

**Table 3:** Analysis of the Al, As, Cd, and Pb concentrations among the femoral cancellous bone, femoral cartilage, anterior cruciate ligament, meniscus, tibial cartilage, tibial cancellous bone and infrapatellar fat pad.

**Parameter**	**Al**	**As**	**Cd**	**Pb**
**Femoral cancellous bone (*n* = 51)**				
AM ± SD	33.74 ± 23.60	2.44 ± 1.46	0.078 ± 0.059	3.22 ± 1.61
Median	26.08	2.23	0.057	2.73
Range	10.18–107.76	0.43–6.53	0.015–0.325	1.34–8.01
**Femoral cartilage (*n* = 51)**				
AM ± SD	33.91 ± 19.52	2.92 ± 1.79	0.124 ± 0.112	2.63 ± 1.48
Median	29.46	2.53	0.083	2.18
Range	11.29–92.36	0.41–8.64	0.026–0.520	0.91–7.18
**Anterior cruciate ligament (*n* = 51)**				
AM ± SD	32.95 ± 15.14	1.76 ± 1.00	0.092 ± 0.087	1.55 ± 0.73
Median	33.01	1.49	0.053	1.34
Range	10.37–96.38	0.39–4.21	0.012–0.432	0.61–3.82
**Meniscus (*n* = 51)**				
AM ± SD	19.84 ± 12.06	4.89 ± 3.25	0.089 ± 0.077	2.21 ± 1.22
Median	16.37	3.85	0.058	1.77
Range	4.91–62.71	0.69–14.77	0.020–0.329	0.49–5.86
**Tibial cartilage (*n* = 51)**				
AM ± SD	34.37 ±2 1.94	3.86 ± 2.58	0.097 ±0.078	3.06 ± 1.58
Median	25.32	3.11	0.081	2.49
Range	12.51–103.62	0.61–11.68	0.015–0.415	0.47–7.38
**Tibial cancellous bone (*n* = 51)**				
AM ± SD	35.44 ± 20.60	3.40 ± 2.12	0.068 ± 0.060	3.30 ± 1.64
Median	30.19	2.89	0.045	2.93
Range	12.09–101.94	0.45–8.95	0.014–0.369	0.86–7.91
**Infrapatellar fat pad (*n* = 51)**				
AM ± SD	49.34 ± 24.22	0.86 ± 0.49	0.043 ± 0.031	0.70 ± 0.41
Median	46.44	0.75	0.033	0.58
Range	15.69–113.59	0.21–2.05	0.006–0.158	0.16–1.98
**Kruskal-Wallis test**				
H	64.05	132.98	43.98	162.82
*p*	<0.01	<0.01	<0.01	<0.01

*AM, arithmetic mean; SD, standard deviation; p, level of significance; n, number of samples; Al, As, Cd, and Pb concentrations were expressed as mg/kg dry weight*.

### Gender and Age

As for the gender, only Cd concentration in the tibial cancellous bone, approximately 60% higher in women than in men, was statistically significantly different. As concentrations in the knee joint structures were higher in men than in women, whereas Pb concentrations were reverse. Despite the noticeable differences in the concentrations of Al, As, and Pb in the studied samples, they were not statistically significant (Table 4). No significant differences were found in the Al, As, Cd, and Pb concentrations between the group up to 65 years and over 65 years.

**Table 4:** Analysis of the Al, As, Cd, and Pb concentrations in the structures of knee joint in men and in women.

**Element**	**Gender**	**Femur**		**ACL**	**Meniscus**		**Tibia**	**IFP**
		**FCB**	**FC**			**TC**	**TCB**	
		**AM ± SD**	**AM ± SD**	**AM ± SD**	**AM ± SD**	**AM ± SD**	**AM ± SD**	**AM ± SD**
		**Median**	**Median**	**Median**	**Median**	**Median**	**Median**	**Median**
		**Range**	**Range**	**Range**	**Range**	**Range**	**Range**	**Range**
Al	Men	33.24 ± 25.12	35.99 ± 20.00	35.70 ± 20.47	21.23 ± 14.66	36.59 ± 23.64	35.83 ± 23.72	48.00 ± 27.41
	(*n* = 16)	22.92	30.94	34.53	17.17	29.40	27.24	40.04
		10.18–98.08	16.28–83.61	13.27–96.38	5.19–62.71	13.52–97.35	15.17–101.94	18.61–113.59
	Women	33.96 ± 23.25	33.96 ± 19.51	31.69 ± 12.12	19.21 ± 10.85	33.35 ± 21.40	35.25 ± 19.38	49.95 ± 23.03
	(*n* = 35)	27.19	26.03	32.37	15.83	25.21	32.15	47.81
		12.58–107.76	11.29–92.36	10.37–54.39	4.91–50.58	12.51–103.62	12.09–98.58	15.69–112.49
	U	NS	NS	NS	NS	NS	NS	NS
	p							
As	Men	2.61 ± 1.63	3.49 ± 2.20	2.00 ± 1.05	5.94 ± 4.05	4.74 ± 3.37	3.79 ± 2.53	0.96 ± 0.56
	(*n* = 16)	2.43	2.76	2.05	4.82	3.50	3.21	0.92
		0.75–6.53	1.34–8.64	0.83–4.17	2.16–14.77	1.63–11.68	0.63–8.95	0.31–2.05
	Women	2.37 ± 1.39	2.66 ± 1.54	1.65 ± 0.97	4.40 ± 2.73	3.46 ± 2.06	3.22 ± 1.91	0.82 ± 0.46
	(*n* = 35)	2.23	2.49	1.46	3.55	2.81	2.79	0.74
		0.43–5.90	0.41–6.63	0.39–4.21	0.69–11.65	0.61–8.27	0.45–7.14	0.21–1.96
	U	NS	NS	NS	NS	NS	NS	NS
	p							
Cd	Men	0.061 ± 0.040	0.102 ± 0.120	0.082 ± 0.067	0.069 ± 0.053	0.083 ± 0.059	0.048 ± 0.036	0.033 ± 0.021
	(*n* = 16)	0.040	0.054	0.051	0.046	0.067	0.037	0.028
		0.021–0.142	0.035–0.520	0.026–0.247	0.025–0.213	0.015–0.219	0.014–0.151	0.006–0.086
	Women	0.086 ± 0.065	0.133 ± 0.109	0.096 ± 0.095	0.098 ± 0.085	0.103 ± 0.086	0.077 ± 0.067	0.047 ± 0.034
	(*n* = 35)	0.059	0.091	0.056	0.067	0.085	0.055	0.037
		0.015–0.325	0.026–0.519	0.012–0.432	0.020–0.329	0.018–0.415	0.017–0.369	0.007–0.158
	U	NS	NS	NS	NS	NS	377	NS
	p						0.049	
Pb	Men	2.65 ± 0.96	2.03 ± 0.64	1.30 ± 0.40	1.88 ± 0.66	2.51 ± 0.66	3.10 ± 1.09	0.63 ± 0.32
	(*n* = 16)	2.44	1.89	1.15	1.65	2.33	2.99	0.57
		1.52–5.30	0.91–3.42	0.71–2.19	1.07–3.33	1.38–4.26	1.31–6.10	0.19–1.57
	Women	3.48 ± 1.78	2.90 ± 1.67	1.66 ± 0.82	2.37 ± 1.38	3.31 ± 1.81	3.40 ± 1.85	0.73 ± 0.45
	(*n* = 35)	2.92	2.52	1.59	1.92	3.12	2.93	0.65
		1.34–8.01	1.06–7.18	0.61–3.82	0.49–5.86	0.47–7.38	0.86–7.91	0.16–1.98
	U	NS	NS	NS	NS	NS	NS	NS
	p							

*FCB, femoral cancellous bone; FC, femoral cartilage; ACL, anterior cruciate ligament; TC, tibial cartilage; TCB, tibial cancellous bone; IFP, infrapatellar fat pad; AM, arithmetic mean; SD, standard deviation; U, Mann-Whitney U test; NS, non-significant difference; Al, As, Cd, and Pb concentrations were expressed as mg/kg dry weight*.

### Place of Residence

**Figure 1:**
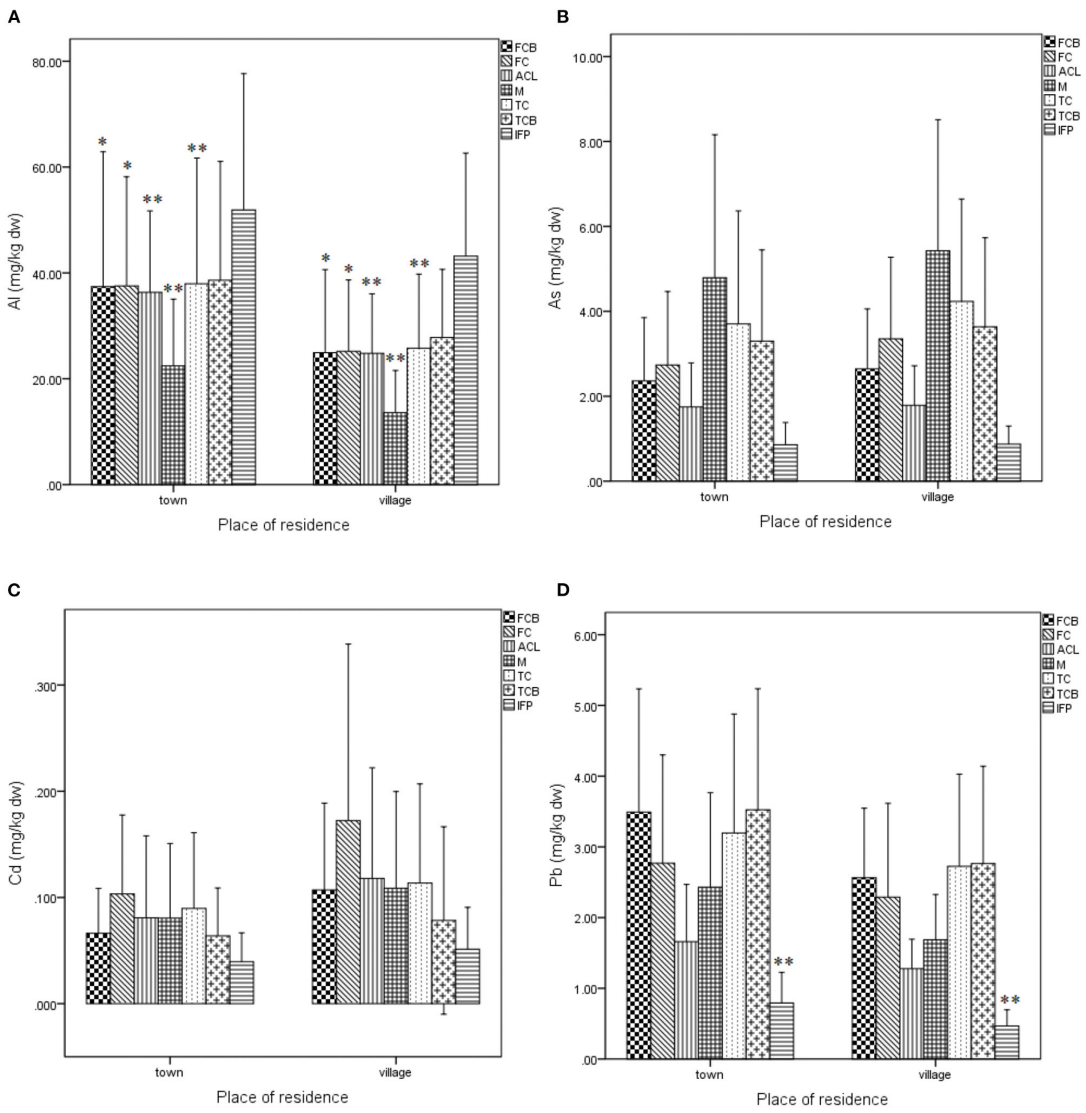
The comparison of the aluminum, arsenic, cadmium, and lead concentrations with residents living in town and village. FCB, femoral cancellous bone; FC, femoral cartilage; ACL, anterior cruciate ligament; M, meniscus; TC, tibial cartilage; TCB, tibial cancellous bone; IFP, infrapatellar fat pad; mg/kg dw, mg/kg dry weight; *p < 0.05; **p < 0.01. **(A)** Comparison of aluminum concentration between urban and rural patients. Al concentrations in the femoral cancellous bone, femoral cartilage, anterior cruciate ligament, meniscus, tibial cartilage in patients living in town were statistically significantly higher than in village at 37.41 vs. 24.93, 37.55 vs. 25.17, 36.34 vs. 24.80, 22.44 vs. 13.60, and 37.95 vs. 25.78 mg/kg dw, respectively. **(B)** Comparison of arsenic concentration between urban and rural patients. **(C)** Comparison of cadmium concentration between urban and rural patients. **(D)** Comparison of lead concentration between urban and rural patients. Pb concentration in the infrapatellar fat pad in patients living in town was statistically significantly higher compared to those residing in village at 0.79 vs. 0.47 mg/kg dw, respectively.

When comparing these toxic element concentrations in the knee joint structures between patients living in town and in village, Al concentrations in the femoral cancellous bone, femoral cartilage, anterior cruciate ligament, meniscus, tibial cartilage in patients living in town were statistically significantly higher than in village at 37.41 vs. 24.93, 37.55 vs. 25.17, 36.34 vs. 24.80, 22.44 vs. 13.60, and 37.95 vs. 25.78 mg/kg dw, respectively (Figure 1). Pb concentration in the infrapatellar fat pad in patients living in town was statistically significantly higher compared to those residing in village at 0.79 vs. 0.47 mg/kg dw, respectively (Figure 1). Cd concentrations in the knee joint structures were higher in patients living in village than in town. Though the observable differences in Cd concentrations in the studied samples, they were not statistically significant (Figure 1).

### Hypertension and Diabetes

The comparisons of these toxic element concentrations in the knee joint structures indicated no significant differences between patients with hypertension (*n* = 20) and with normal blood pressure (*n* = 31). Al and Cd concentrations were higher in normotensive participants than in hypertensive subjects. Taking into consideration diabetes, only As concentration in the tibial cancellous bone had statistically significant difference (U = 372, *p* = 0.035), approximately 41% higher in diabetics than in non-diabetics at 4.27 and 3.03 mg/kg dw, respectively. No significant differences in the concentrations of Al, Cd, and Pb in the knee joint structures between diabetic patients and non-diabetic patients were found.

### Spearman's Correlation Coefficient

Statistically significant Spearman's positive correlation coefficients were observed between Al and Pb in the knee joint structures (Table 5).

**Table 5:** Spearman's correlation coefficients between Al and Pb in the analyzed structures of knee joint.

	**FCB**		**FC**		**ACL**		**M**		**TC**		**TCB**		**IFP**	
	**Al**	**Pb**	**Al**	**Pb**	**Al**	**Pb**	**Al**	**Pb**	**Al**	**Pb**	**Al**	**Pb**	**Al**	**Pb**
FCB	Al														
	Pb	0.40^**^													
FC	Al		0.50^**^												
	Pb	0.41^**^		0.44^**^											
ACL	Al		0.41^**^		0.39^**^										
	Pb	0.33^*^		0.44^**^		0.36^**^									
M	Al		0.53^**^		0.52^**^		0.43^**^								
	Pb	0.43^**^		0.48^**^		0.42^**^		0.49^**^							
TC	Al		0.43^**^		0.40^**^		0.41^**^		0.51^**^						
	Pb	0.41^**^		0.45^**^		0.42^**^		0.50^**^		0.40^**^					
TCB	Al		0.34^*^		0.32^*^		0.34^*^		0.35^*^		0.36^**^				
	Pb	0.40^**^		0.52^**^		0.40^**^		0.50^**^		0.44^**^		0.41^**^			
IFP	Al		0.32^*^		NS		0.33^*^		0.39^**^		0.32^*^		0.34^*^		
	Pb	0.33^*^		0.42^**^		0.29^*^		0.45^**^		0.37^**^		0.31^*^		0.32^*^	

*FCB, femoral cancellous bone; FC, femoral cartilage; ACL, anterior cruciate ligament; M, meniscus; TC, tibial cartilage; TCB, tibial cancellous bone; IFP, infrapatellar fat pad; NS, non-significant;*

* *p < 0.05;*

** *p < 0.01*.

## Discussion

The accumulation and retention of toxic elements may have adverse effects on the health by replacing other elements necessary for normal metabolism and interfering with homeostasis in the organism (8). Joint damage caused by the toxic elements may occur after many years of exposure or may be sudden. It is becoming increasingly urgent to assess the risk of joint tissue exposed to toxic elements from environmental pollution (31). Due to slow remodeling and low metabolic rate, knee joint structures, including meniscus and anterior cruciate ligament, can serve as good biomarkers for long-term toxic element accumulation resulting from environmental or occupational exposure (7, 44).

In this study, these toxic element concentrations in almost all of the knee joint structures can be arranged in the following ascending series: Cd <Pb <As <Al (Table 3). Al concentration was the highest in the south part of Poland (35) and the lowest in Kaluga Region, Russia (7). Some authors reported that Cd concentration in the cancellous bone usually varied from 0.014 mg/kg dw to 0.94 mg/kg dw (Table 1). Cd concentration in this study was lower or higher than their results (3, 5, 7, 29, 31, 34, 36, 38, 39). The highest Cd concentration was observed in patients inhabiting in urban areas of the Upper Silesian Industrial Zone, Poland (29), while the lowest was noted in the Kragujevac region, Serbian (3). Various outcomes were found in the studies about the residents from the different counties, demonstrating that the levels of toxic elements were related to the geographic areas. When compared with the concentrations of ribs, Al, Cd, and Pb concentrations in the tibial cancellous bone are higher in this study (7). According to the study by Stojsavljević et al. (3), the femoral head shows higher As concentration and lower concentrations of Cd and Pb as compared to this study. These findings indicate that the bone sites may differ in the content of toxic elements.

Interactions between toxic elements can disturb the homeostasis in the organisms, which can cause their toxic effects (33). Lanocha-Arendarczyk et al. (38) found significant positive correlation between Pb and Cd in the tibial plateau, which was consistent with the study conducted by Brodziak-Dopierala et al. (44). This study also observed the positive correlations between Cd and Pb in the knee joint structures without significant difference. Based on the conducted studies, significant positive correlation between Pb and Al in the knee joint structures was found. Positive quantitative correlations between Pb and Cd, as well as Pb and Al in the knee joint structures probably arise from a common environmental origin of the elements (45).

Some studies have indicated that the toxic element concentrations in the joint are influenced by gender (8, 38). Babuśka-Roczniak et al. (35) indicated that women had more Al concentrations in the tibial cancellous bone and meniscus than men. Nevertheless, our study found higher Al concentrations in the tibial cancellous bone and meniscus of men compared to women. Roczniak et al. (39) noticed that Cd concentration in the tibial cancellous bone in women was lower than in men (0.015 vs. 0.019 mg/kg dw), which was similar to the study by Lanocha-Arendarczyk et al. (38). However, we observed significantly higher Cd concentration in tibial cancellous bone of women as compared to men. The tendency of a greater Cd accumulation in women was often cited in the literature (29, 46), which can be due to the promotion of gastrointestinal absorption of Cd under low Fe stores (47). This finding may be the primary reason why itai-itai disease mainly influences women (44). Kosik-Bogacka et al. (33) noticed that Pb concentrations in the anterior cruciate ligament, meniscus and cartilage were higher in men than in women, while significant difference of Pb concentration between men and women only existed in meniscus. Roczniak et al. (40) noted significantly higher Pb concentrations in the femur, tibia and meniscus of men (3.22, 3.99, and 0.53 mg/kg dw, respectively) compared to women (2.41, 2.16, and 0.24 mg/kg dw, respectively). However, this study found higher Pb concentrations in the knee joint structures of women as compared to men. This finding may be because the toxic element concentrations in the female body are affected by hormonal changes occurring during menstruation, pregnancy or menopause (33, 48). Low Fe concentration in women will promote the Cd absorption in the gastrointestinal tract (47), and a synergistic correlation between Cd and Pb is observed (45). Hence, the concentrations of Cd and Pb in the body are generally higher in women than in men (39).

Age is associated with the occurrence and deposition of toxic elements in bone (35). Some toxic elements may increase with age in bone (7, 37). Zaichick et al. (7) demonstrated that the concentrations of Al, Pb, and Cd in the ribs in the group over 35 years old were higher than in the group up to 35 years old, but no significant differences between the two age groups were found. Taking into account Cd, Chang et al. (49) indicated that Cd concentration in bone increased with age. Nevertheless, Cd concentration between the two age groups was nearly at the same level in this study, thereby confirming the result of Roczniak et al. (39). It is suggested that Cd storage in the human body is constantly exchanged and no long-term accumulation is present (39). Kosik-Bogacka et al. (33) noted that Pb concentration in the cartilage was positively correlated with increasing age, which was similar to the studies by Jurkiewicz et al. (32) and Brodziak-Dopierała et al. (30). Zaneta et al. (41) observed no significant difference in Pb concentration in the infrapatellar fat pad between the groups aged 56–74 and 75–87 years. We also found no significant difference in Pb concentration in the knee joint structures between the group up to 65 years and over 65 years. These findings mean that Pb concentration in bone remains at a stable level after adulthood, which is of great significance to bone metabolism and human health (49).

The differences in the toxic element concentrations between urban and rural residents can reflect the degree of environmental pollution (50). Based on our conducted studies, Pb concentration in the infrapatellar fat pad in town was significantly higher compared to village. However, Zaneta et al. (41) indicated that Pb concentration in the infrapatellar fat pad was higher in rural areas than in urban areas without significant difference. The differences between the obtained values might result from the size and diversity of the studied samples. Pb isotope compositions show that Pb pollution is caused by incinerator ash, gasoline Pb and building materials, indicating that the atmospheric deposition is the main source. Pb pollution may be more serious in urban areas than in rural areas (51). This finding is similar to the results of this study.

Toxic elements are considered as risk factors for cardiovascular disease (52, 53). Zhang et al. (52) indicated that Al could induce hypertension, which may be caused by the dysfunction of erythrocyte membrane after acute or chronic Al exposure. Schmidt et al. (54) found the positive association between circulating plasma Al levels and hypertension. Abhyankar et al. (55) noticed that As resulted in hypertension through oxidative stress and nitric oxide inhibition. Hall et al. (56) observed a significant positive correlation between As concentration in the blood and the prevalence of hypertension. Da Cunha Martins et al. (57) demonstrated that Cd could increase the risk of hypertension through sodium retention and volume overload due to the renal tubules injuries caused by Cd. Miao et al. (58) found a positive correlation between blood Pb level and hypertension. Nevertheless, no significant differences in the effect of hypertension on the concentrations of Al, As, Pb, and Cd were observed in this study. Kosik-Bogacka et al. (33) also found no significant impact of hypertension on Pb concentration in the knee joint.

### Strengths of the Study

To the best of our knowledge, this study is the first comprehensive determination of the Al, As, Cd, and Pd concentrations in the femoral cancellous bone, femoral cartilage, anterior cruciate ligament, meniscus, tibial cartilage, tibial cancellous bone and infrapatellar fat pad simultaneously, which can provide a basis for the establishment of reference values of toxic elements in the knee joint structures and is helpful to the assessment of environmental pollution caused by toxic elements. Additionally, the relationships between relevant factors such as gender, age, place of residence, hypertension, diabetes and the toxic elements, which have guiding significance for the prevention and control of adverse health caused by toxic elements, have been demonstrated in this study, thereby improving public health.

### Limitations of the Study

This study has several limitations. First, 51 patients participated in this study. Such an insufficient sample size can preliminarily explore the relationships between the toxic element concentrations and age, gender, place of residence, hypertension and diabetes, and support the conclusion of this study in the knee joint structures. In the follow-up research, we will collect more samples to investigate the influence of relevant factors on the toxic element concentrations in the knee joint structures, thereby resulting in more significant meaningful conclusions. Second, occupational exposure is one of the sources of toxic element accumulation. The utilization of these toxic elements in various industries, such as color pigments and alloys, will lead to occupational exposure (59). However, no qualified samples were collected in this study because of the limited number of patients with occupational exposure to toxic elements. Hence, this study does not involve occupational exposure. In the future, we will continue to collect samples of patients with a history of occupational exposure to toxic elements. Finally, this study explored the relationships between Al, As, Cd, and Pb concentrations and relevant factors such as age, gender, place of residence, hypertension and diabetes. However, other factors also affect the accumulation of toxic elements. Thus, the relationships between these toxic element accumulation and related factors such as food, drinking water, air, alcohol and tobacco should further establish, and the pollution data in the studied regions must be mastered to find more influencing factors in the follow-up studies.

## Conclusion

The obtained results of the investigated toxic elements may serve as a basis for establishing reference values of Al, As, Cd, and Pb for the femoral cancellous bone, femoral cartilage, anterior cruciate ligament, meniscus, tibial cartilage, tibial cancellous bone and infrapatellar fat pad of patients with knee degenerative diseases. Analysis of the relationships between relevant factors and the toxic elements in the knee joint demonstrated the effect of gender, age, place of residence, hypertension and diabetes. Furthermore, the correlations between Al and Pb were found. These findings have guiding significance for the prevention and control of adverse health caused by toxic elements, thereby improving public health.

## Data Availability Statement

The original contributions presented in the study are included in the article/supplementary material, further inquiries can be directed to the corresponding author/s.

## Ethics Statement

The studies involving human participants were reviewed and approved by the Ethics Committee of the Sixth People's Hospital Affiliated to Shanghai Jiaotong University, Shanghai, China. The patients/participants provided their written informed consent to participate in this study. Written informed consent was obtained from the individual(s) for the publication of any potentially identifiable images or data included in this article.

## Author Contributions

XY, TC, and GL designed the research and conducted the experiments. CX, RM, and WX analyzed the data and drafted the manuscript. All authors contributed to the article and approved the submitted version.

## Conflict of Interest

The authors declare that the research was conducted in the absence of any commercial or financial relationships that could be construed as a potential conflict of interest.

## Publisher's Note

All claims expressed in this article are solely those of the authors and do not necessarily represent those of their affiliated organizations, or those of the publisher, the editors and the reviewers. Any product that may be evaluated in this article, or claim that may be made by its manufacturer, is not guaranteed or endorsed by the publisher.
